# Performance of Antipseudomonal β-Lactams on the Accelerate PhenoTest BC Kit against a Collection of Pseudomonas aeruginosa Isolates

**DOI:** 10.1128/JCM.01781-20

**Published:** 2021-01-21

**Authors:** Aaron Sikorski, Alena Shamsheyeva, Dulini Gamage, Niels Oppermann, Amira A. Bhalodi, Romney M. Humphries

**Affiliations:** a Accelerate Diagnostics, Inc., Tucson, Arizona, USA; Medical College of Wisconsin

**Keywords:** *Pseudomonas aeruginosa*, β-lactams, susceptibility testing

## LETTER

Determining phenotypic antimicrobial susceptibilities of Pseudomonas aeruginosa is particularly valuable due to the complexity of resistance mechanisms this organism can harbor. The Accelerate PhenoTest BC kit (AXDX) provides a fast phenotypic antimicrobial susceptibility testing (AST) method for testing P. aeruginosa directly from positive blood culture. This study evaluated updates to the Accelerate PhenoTest BC kit made in order to improve the performance of beta-lactams when tested against P. aeruginosa (1, 2).

One hundred forty-four P. aeruginosa isolates were used to spike a blood culture bottle containing healthy donor blood and incubated until positivity. Aliquots of positive blood culture were tested on the Accelerate Pheno system (software 1.4.1.25) as previously described (3). AST was also performed in triplicate by CLSI reference broth microdilution (BMD) using isolated colonies (4). MIC results were compared to BMD results to calculate essential agreement (EA), categorical agreement (CA), and rates of very major (susceptible by AXDX, resistant by reference), major (resistant by AXDX, susceptible by reference), and minor (intermediate by one AST method, susceptible or resistant by the other method) errors (5). For EA, BMD results were truncated to the same range as those reported by the Accelerate Pheno system. FDA and CLSI breakpoints were applied (Table 1) (6, 7).

**TABLE 1:** Current FDA- and CLSI-designated breakpoints of antipseudomonal beta-lactams

Beta-lactam antibiotic	Breakpoint (μg/ml)		
	Susceptible	Intermediate	Resistant
Aztreonam (FDA and CLSI)	≤8	16	≥32
Cefepime (FDA)	≤8		≥16
Cefepime (CLSI)	≤8	16	≥32
Ceftazidime (FDA)	≤8		≥16
Ceftazidime (CLSI)	≤8	16	≥32
Meropenem (FDA and CLSI)	≤2	4	≥8
Piperacillin-tazobactam (FDA and CLSI)	≤16/4	32/4–64/4	≥128/4

Table 2 provides the EA, CA, and error rates for the isolates tested on both the updated and previous assays. With respect to the updated assay and when interpreted by FDA breakpoints, nine of 11 errors observed for cefepime were within EA, including the single very major error. Cefepime and ceftazidime do not have an intermediate interpretation by FDA breakpoints; therefore, all errors can be classified only as major or very major for these antimicrobials (6). When interpreted with CLSI breakpoints, all cefepime errors were minor and 17/21 errors were within EA. Bias toward a more resistant MIC for cefepime was observed by AXDX (Table 3). High cefepime minor-error rates with P. aeruginosa have been observed in various studies with other automated platforms, such as Vitek2 (9 to 18%), MicroScan WalkAway (32% to 48%), and BD Phoenix (18%) (8–11). When results were interpreted by FDA breakpoints, a total of five errors were observed with ceftazidime, and 2 of the 3 very major errors were within EA, a good case example demonstrating the challenges of interpreting errors when an intermediate breakpoint does not exist. When results were interpreted with CLSI breakpoints, 1 major and 1 very major error remained for ceftazidime, with EA and CA above 90%. Fifteen minor errors (10.4%) were observed with meropenem (Table 2), among which 9 were within EA. Eleven of the minor errors were due to the MIC being interpreted as resistant by AXDX but intermediate by BMD.

**TABLE 2:** Performance of antipseudomonal beta-lactams tested against P. aeruginosa isolates on the Accelerate PhenoTest BC kit compared with BMD

Beta-lactam antibiotic^*a*^	No. of isolates^*b*^			No. (%) with agreement		No. (%) of errors		
	Total	S	R	CA	EA	Very major	Major	Minor
Aztreonam (FDA and CLSI)*	144	105	35	134 (93.1)	135 (93.8)	0 (0)	1 (1.0)	9 (6.2)
Aztreonam (FDA and CLSI)	144	105	35	122 (84.7)	124 (86.1)	0 (0)	1 (1.0)	21 (14.6)
Cefepime (FDA)*	143	107	36	132 (92.3)	136 (95.1)	1 (2.8)	10 (9.3)	
Cefepime (FDA)	144	108	36	84 (58.3)	81 (56.2)	0 (0)	60 (55.6)	
Cefepime (CLSI)*	143	107	29	122 (85.3)	136 (95.1)	0 (0)	0 (0)	21 (14.7)
Cefepime (CLSI)	144	108	29	76 (52.8)	81 (56.2)	0 (0)	2 (1.9)	66 (45.8)
Ceftazidime (FDA)*	141	103	38	136 (96.5)	136 (96.5)	3 (7.9)	2 (1.9)	
Ceftazidime (FDA)	144	104	40	46 (31.9)	47 (32.6)	0 (0)	98 (94.2)	
Ceftazidime (CLSI)*	141	103	31	132 (93.6)	136 (96.5)	1 (3.2)	1 (1.0)	7 (5.0)
Ceftazidime (CLSI)	144	104	33	40 (27.8)	47 (32.6)	0 (0)	20 (19.2)	84 (58.3)
Meropenem (CLSI and FDA)*	144	102	25	127 (88.2)	136 (94.4)	0 (0)	2 (2.0)	15 (10.4)
Meropenem (CLSI and FDA)	144	102	25	98 (68.1)	107 (74.3)	0 (0)	2 (2.0)	44 (30.6)
Piperacillin-tazobactam (CLSI and FDA)*	138	101	30	130 (94.2)	133 (96.4)	0 (0)	0 (0)	8 (5.8)
Piperacillin-tazobactam (CLSI and FDA)	144	106	31	45 (31.2)	52 (36.1)	0 (0)	12 (11.3)	52 (36.1)

a An asterisk indicates that the improved software was used.

b S, susceptible; R, resistant.

**TABLE 3:** Error trends of beta-lactam antibiotics tested against P. aeruginosa isolates on Accelerate PhenoTest BC kit compared with BMD

Beta-lactam antibiotic (no. of errors)	No. of results		
	More susceptible	More resistant	Within EA
Aztreonam (10)	6	4	9
Cefepime (11)	1	10	10
Cefepime (CLSI) (21)	9	12	17
Ceftazidime (5)	3	2	2
Ceftazidime (CLSI) (9)	3	6	6
Meropenem (17)	1	16	9
Piperacillin-tazobactam (8)	1	7	6

Overall, the most notable improvements with the updated assay are within the major and minor error rates. In the original clinical trial data set for the Accelerate Pheno system, a total of 43 major errors were observed among the Gram-negative organisms, with 26% of them being for beta-lactams tested against P. aeruginosa. This resulted in major-error limitations imposed by the FDA and the aim for the updates to the assay described herein (1). The data presented here are from a different population of isolates than those used in the original clinical trial. Specifically, the current data set was enriched to include approximately 20% of isolates with MICs at the breakpoint, allowing a robust evaluation of performance postimprovement. Furthermore, the population described here is approximately 10% less susceptible than what is likely to be observed in clinical laboratories based on U.S. surveillance of P. aeruginosa bloodstream infections (12). This is important, as differences in MIC distributions impact the propensity of errors. Therefore, direct comparisons between two different isolate sets, such as the present data and that described by Pancholi et al. (1), cannot be directly made. Nonetheless, the improvements described herein led to the removal of major-error limitations for piperacillin-tazobactam, meropenem, ceftazidime, and cefepime.

P. aeruginosa susceptibility testing is known to be challenging (8–11). As technologies for susceptibility testing advance, development of assays for these difficult-to-test organisms is prudent and likely an ongoing necessity. Moreover, clinical microbiology labs should seek to understand their local epidemiology when evaluating an assay, as performance can vary among different populations of isolates. These data demonstrate markedly improved performance, particularly with respect to major errors, of beta-lactams against P. aeruginosa on the Accelerate Pheno system compared with previous versions of the assay.
